# Preparation of Lauroyl Grafted Alginate-Psyllium Husk Gel Composite Film with Enhanced Physicochemical, Mechanical and Antimicrobial Properties

**DOI:** 10.1038/s41598-018-35632-9

**Published:** 2018-11-21

**Authors:** Clara Fernandes, Pratap Chandra Acharya, Shikha Bhatt

**Affiliations:** 1 Shobhaben Pratapbhai Patel School of Pharmacy and Technology Management, SVKM’S NMIMS, Mumbai, 400056 India; 2 Department of Pharmacy, Tripura University (A Central University), Suryamaninagar, 799022 Tripura (W), India

**Keywords:** Drug delivery, Pharmaceutics

## Abstract

In this study, a lauroyl grafted hydrophobic glycolipid derivative of alginate has been successfully synthesized and characterized. This glycolipid has been incorporated into *Psyllium* husk gel-alginate composite films and compared with the films containing only Psyllim husk gel and Psyllim husk gel-alginate for its mechanical and physicochemical properties. Additionally, the composite film has also been evaluated for protein adsorption and antimicrobial property to verify its utility in biomedical applications. The results showed that the composite films have enhanced physicochemical and mechanical properties. The film produced better swelling characteristic and lower protein adsorption property indicating the usefulness of the film in wound care dressing, particularly for low suppurating wounds. Incorporation of the synthesised glycolipid derivative also imparts antimicrobial activity to the composite film. Therefore, the developed film is capable of sustaining the microbial contamination during the storage and also valuable in the biomedical utility including wound dressings.

## Introduction

Composites are hybridised materials with more than two components and have different material properties than their individual components. Polysaccharide composites are considered to be more ecological due to their biodegradable nature^1^. For this reason the polysaccharide composites have found their applications in the fields of medical, paper, food, textile, packing, electronic and mechanical engineering. These composites are often characterised for their mechanical, gelling, film-forming, water-vapor permeability and antimicrobial properties^2^.

Sodium alginate is a natural biopolymer obtained from several species of brown seaweed^3^. Chemically, alginate is a linear unbranched polysaccharides containing (1 → 4)-linked β-D-mannuronate and α-L-guluronate residues respectively, in different sequences^4^. Although sodium alginate has found its use in several biomedical applications, the scopes of its future application are hindered due to the limiting factors such as the strong hydrophilic character, low thermal stability and poor mechanical strength. For this reason, several approaches have been made to improve the properties of sodium alginate by compounding it with other polymers^5^.

On the other hand, the husk obtained from the seeds of the plant *Plantago ovate*, commonly known as *Psyllium*, is grown all over the world as a major seasonal crop because of its uses in pharmaceuticals, cosmetics and food products. These applications are attributed to the demulcent, laxative, emollient and adsorbent properties of the mucilage present in the husk^6,7^. In contrast to sodium alginate, *Psyllium* is hydrophobic in nature and well known for its gelling capabilities, swelling ability and water uptake properties. These properties are primarily credited to the presence of hyper branched acidic arabinoxylan backbone with both (1–4) and (1–3) linkages as well as high percentage of hemicelluloses^8^.

Both, alginate and *Psyllium* are known to absorb water/body fluids multiple times of its own weight^9^. For this reason, composite of alginate has been prepared by incorporating it in the *Psyllium* mucilage in order to generate ultra high absorbent hybrid with interpenetrating network, particularly for the wound management and probiotic benefits^6,10,11^. However, the effect of alginate-*Psyllium* composites on film formation and its physicochemical properties has not been studied.

Therefore, in order to improve the film forming capabilities of alginate, we have synthetically grafted lauric acid on alginate to yield a hydrophobic glycolipid derivative. The glycolipid derivative has been incorporated in to the *Psyllium* husk mucilage to form composite film in presence of ZnCl_2_ as the cross linker. The hydrophobically modified alginate-*Psyllium* composite film has been optimized and evaluated for its mechanical and physicochemical properties such as thickness, weight variation, folding endurance, percentage elongation, moisture content, swelling studies, pH studies and moisture vapour transmission rate. In addition, the composite film has also been evaluated for protein adsorption study to predict wound adherence and antimicrobial property in order to verify the reduction in the microbial load of the prepared film.

## Results and Discussion

Various additional experimental results are compiled and provided as supplementary data as well as Supplementary Tables S1–S16 and Supplementary Figs S1–S9.

### Synthesis of lauric acid grafted alginate (glycolipid)

Future scopes of sodium alginate in biomedical applications have been impeded due to the limiting factors such as the strong hydrophilic character, low thermal stability and poor mechanical strength. Therefore, it was envisaged to synthetically modify the alginate to its hydrophobic congener by conjugating it with lipophilic counterparts such as fatty acid to yield a hydrophobic glycolipid. At the same time, it was also thought to prepare the glycolipid in such a way that it would impart enhanced hydrophobicity, mechanical strength along with strong antibacterial properties to wade off the microbial contaminations on its own. Lauric acid is known as the mightiest antimicrobial fatty acid^12,13^. In recent past, attempts have also been made to prepare amphiphilic lauryl-alginate conjugates. However, the physicochemical characterization has not proved any beneficial effect or biomedical implications^14,15^.

**Figure 1:**
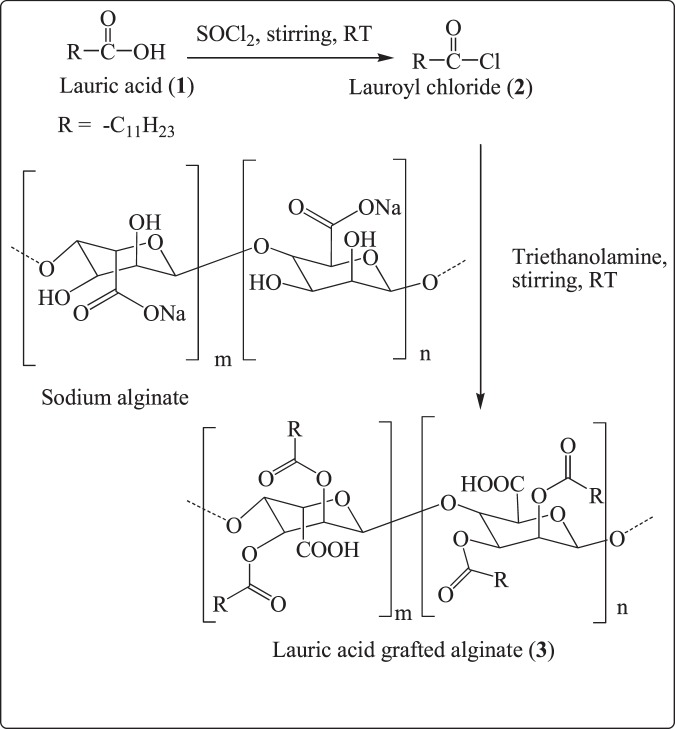
Scheme for the synthesis of lauric acid grafted glycolipid (**3**).

For this reason, the lauric acid has been grafted on to the hydrophilic –OH functions of alginate to produce a hydrophobic glycolipid. The synthetic approach is depicted in Fig. 1. Initially, the lauric acid was converted to lauroyl chloride using thionyl chloride. The excess of lauroyl chloride was reacted with a solution of sodium alginate in triethanolamine to form the glycolipid. The crude glycolipid was obtained as a jelly mass and purified from the excess of lauroyl chloride and triethanolamine by repeated washings with water and subsequent vacuum filtration. It was dried in vacuum desiccator to get pure solid glycolipid derivative. The purity was ascertained using Lassigne’s fusion extract test in triplicates for residual nitrogen from triethanolamine. The negative test for nitrogen indicated that the glycolipid is free from the trientanolamine byproducts.

The DSC (Differential Scanning Calorimetry) thermogram of the glycolipid **3** (Supplementary Fig. S3) showed exothermic peak at 173.64 °C which further confirmed the formation and purity of the synthesised glycolipid. The appearance of single exotherm at a higher heat of crystallization indicates that the product is free from residual lauroyl chloride, its hydrolysed products or any other starting materials (Supplementary Fig. S3).

The complete grafting of the lauroyl function on the –OH groups of alginate was confirmed from the FTIR (Fourier Transform Infrared Spectroscopy) spectrum of the glycolipid **3** (Fig. S4) by comparing it with that of the starting alginate (Supplementary Fig. S1). In the parent alginate, a broad IR (Infrared) absorption band at wavenumber of 3402 cm^−1^ was observed due to the free hydroxyl function of the parent alginate. The band at 1090 and 1031 cm^−1^ were observed due to C–O–C stretching vibration of the saccharide structure. The strong peak at 1618 cm^−1^ was assigned to the stretching vibrations of carboxyl groups.

Whereas, appearance of a sharp peak at a lower wavenumber of 3313 cm^−1^ in the FTIR spectrum of the synthesised glycolipid indicated the presence of –OH stretch of carboxylic acid and confirmed the complete disappearance of free hydroxyl groups of alginate due to successful grafting of the lauroyl function onto it. This was further authenticated by the appearance of carbonyl stretch of the newly formed ester bond of the glycolipid and the free carboxylic acid as a merged broad peak at a higher wavenumber of 1711 cm^−1^ .The FTIR spectrum also featured additional peaks at 2922 cm^−1^ due to methylene groups of lauroyl function.

To find out the degree of lauroyl substitution on the alginate, the synthesised glycolipid **3** was subjected to ^1^H NMR (Nuclear Magnetic Resonance) study. However, the hydrophobic glycolipid showed poor solubility in several NMR solvents and a good spectrum could not be obtained. This is also known to be common problem for substituted polysacharides for the determination of degree of substitution using NMR^16^. However, the complete disappearance of the –OH peak at 3402 cm^−1^ in the FTIR spectrum of the glycolipid suggested the total substitution of lauroyl group on alginate^17,18^.

### Extraction of Psyllium husk mucilage and characterization

The *Psyllium* husk mucilage was extracted from the husk by adopting suitable modification to the approach described previously^6^. Three methods of mucilage extraction namely hot, cold and acid-base methods were employed. The hot method of extraction produced maximum amount of mucilage compared to other method of extraction. Therefore, the hot method of extraction was utilised to obtain the *Psyllium* husk mucilage and was characterized for its viscosity and the nature of carbohydrates present in the gel (Supplementary Table S5).

The *Psyllium* husk gel extracted by the hot extraction method was subjected to rheology studies before and after sterilization in different concentrations (1, 2, 3, 4, 5 and 6% w/v) of gel. The moist heat sterilization was used to understand the rheological behaviour of the gel on heat sterilization and the results are summarised in Supplementary Table S6 and represented in Supplementary Fig. S5. The viscosity was found to increase with the concentration of the gel both before and after the sterilization. The increase in viscosity at higher concentrations is attributed to the formation of increased hydrogen bonds between the polysaccharides and water leading to a very rigid structure^19^. However, the viscosity decreased when the RPM (Rotation Per Minute) of the spindle was increased. This could be due to the reason that for a given viscosity, the viscous drag or resistance to flow is proportional to the spindle’s speed of rotation, size and shape. Also, it was observed that the viscosity of gel decreases after sterilization at all RPM, indicating changes and breakdown in the molecular structure of the polysaccharides present in the gel. A 2% w/v gel was also tested for the different types of carbohydrates present in the *Psyllium* husk (provided in supplementary characterization data Table S5). The results showed presence of arabinose, sucrose and glucose in the gel.

### Preparation of the hydrophobically modified alginate-Psyllium husk composite films and evaluation of physicochemical, mechanical and performance parameters

The *Psyllium* husk gel was casted into films either alone or incorporating sodium alginate and the synthesised glycolipid derivative of alginate to obtain the composite film. Several batches were prepared and the conditions were optimised for film preparation.

Firstly, films were prepared using only *Psyllium* husk gel to observe the effect of concentration on film formation. The optimization results are shown in Supplementary Table S7. A 4% or 5% w/v gel was found to be most suitable for the film formation. The % moisture content was found to increase with increase in concentration (Supplementary Fig. S6a). The drying temperature was also investigated ranging from 60–80 °C keeping the drying time constant for 3 hrs. The optimum temperature for the film formation was recorded to be 80 °C for films with concentration of 4% or 5% w/v. The *Psyllium* husk gel films were evaluated for their physicochemical and mechanical properties such as thickness, weight variation, folding endurance and percentage elongation (Supplementary Table S8) and the films containing 4% or 5% w/v concentration showed optimum results. Therefore, the 4% w/v gel concentration was fixed for the composite film formation.

Secondly, composite films were prepared using a fixed concentration of 4% w/v *Psyllium* husk gel and varied concentrations of sodium alginate (0.25, 0.5, 0.75, 1, 1.25 and 1.5% w/v) in order to study the effect on film formation and physicochemical properties. Optimization of the films was done by evaluating them for their physicochemical and mechanical parameters such as such as thickness, weight variation, folding endurance and percentage elongation (Supplementary Table S9) along with performance parameters such as swelling studies and *in vitro* deformation (Supplementary Table S10)^20^.

**Figure 2:**
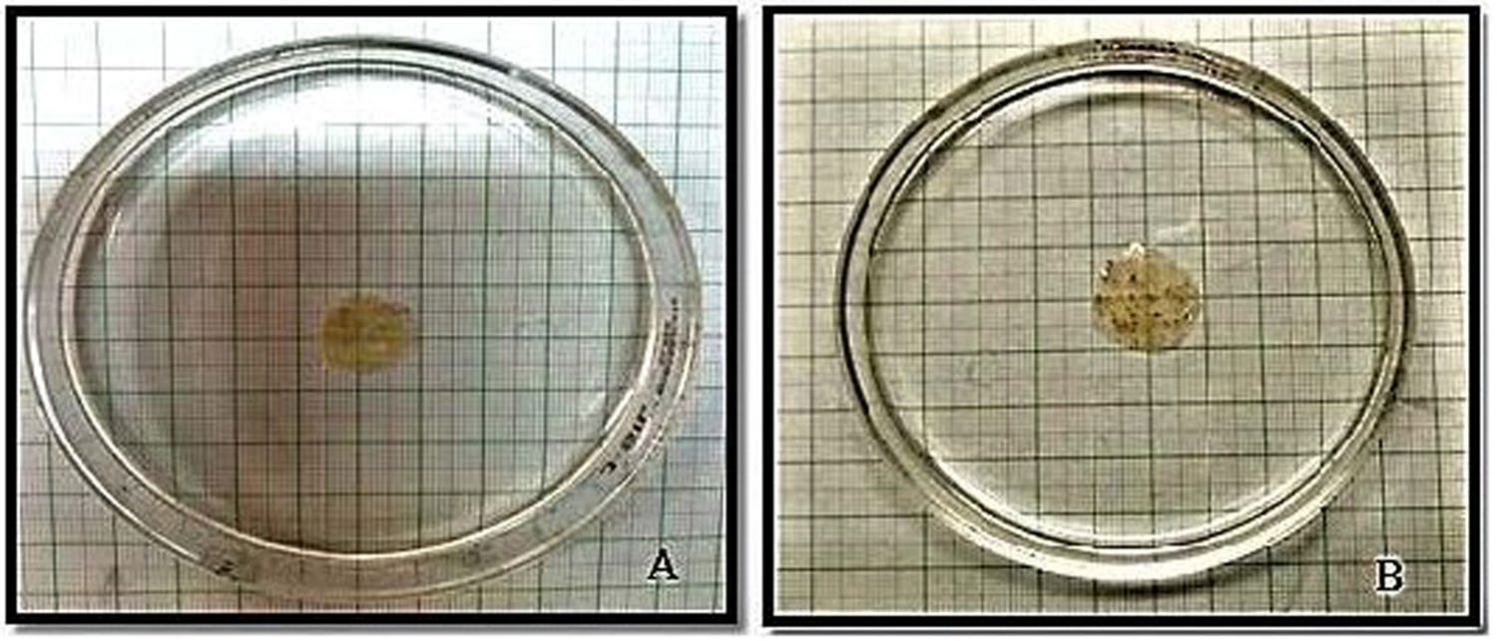
Swelling studies of film containing 4.75% w/v Psyllium husk gel and sodium alginate (**A**) = at 0 min and (**B**) = at 1 hr.

From the results obtained from the evaluation of films for various physicochemical and mechanical parameters such as thickness, weight variation, folding endurance and percentage elongation it was found that film with concentration 4.75% w/v showed optimum results. The films displayed greater swelling at higher concentration when placed in buffer solution. However, the film with 4.75% w/v concentration showed optimum results in all physicochemical and mechanical parameters including the swelling study (Fig. 2). Thus for further evaluation, film with concentration of 4.75% w/v was selected. Although, addition of sodium alginate to the *Psyllium* husk gel did not cause marked difference in the physicochemical and mechanical properties of the film but the films did not stay sticky to the petri plates any longer. This property can be attributed to the tendency of sodium alginate to form tiny pores into the film.

**Figure 3:**
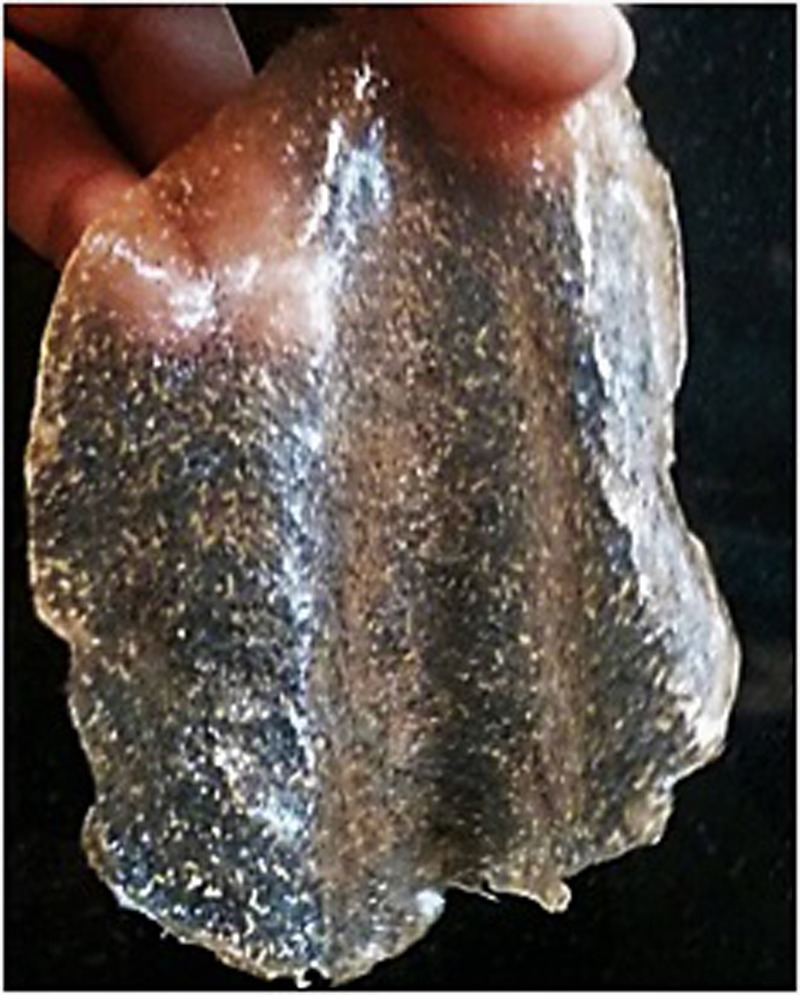
Picture of a lauroyl grafted alginate-Psyllium husk gel non-adhesive composite film.

**Figure 4:**
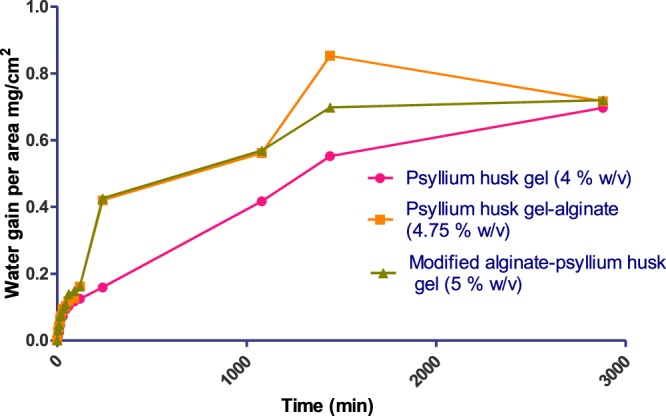
Comparison of MVTR of the optimized films.

Finally, a composite film was prepared by incorporating the lauric acid grafted alginate to the above mentioned *Psyllium* husk gel-alginate film by solvent casting method. The physical cross linking by zinc chloride contributed to the improvement in the mechanical property of the film. The glycolipid loaded composite film (Fig. 3) was evaluated for physicochemical and mechanical properties such as thickness, weight variation, folding endurance and percentage elongation and performance properties such as swelling studies, *in vitro* deformation studies (Table 1), moisture vapour transmission rate (Supplementary Table S11 and Fig. 4), loss on drying (Supplementary Table S12, Supplementary Fig. S6b) and effect of *pH* change (Supplementary Tables S13, S14, Supplementary Figs S7 and S8). These parameters were also compared with films made from only *Psyllium* husk gel and the *Psyllium*-alginate combination taking them as controls.

**Table 1:** Physicochemical and mechanical evaluations of hydrophobically modified alginate-psyllium husk gel composite film.

Concentration (in % w/v)	Thickness (in mm) study in triplicate	Weight variation (in g) study in triplicate	Folding endurance (no. of times)	% Elongation study in triplicate	% Swelling study in triplicate	*In vitro deformation* (in min) study in triplicate
4% Psyllium gel	1.40 ± 0.1	0.035 ± 0.002	128 ± 0.01	6.32 ± 0.03	NA	NA
4.75 (4% Psyllium gel + 0.75% alginate)	0.35 ± 0.02	0.472 ± 0.01	135.33 ± 0.57	24.28 ± 0.02	14.30 ± 0.02	5.5 ± 0.01
5 (4% Psyllium gel + 0.75% alginate + 0.25% lauryl grafted alginate)	0.37 ± 0.02	0.481 ± 0.001	137 ± 0.01	27.28 ± 0.02	15.39 ± 0.03	5.9 ± 0.05

The incorporation of the lauroyl grafted alginate in to the *Psyllium* husk gel-alginate composite film did not affect the physicochemical or mechanical properties indicating that uniform and desirable films can be prepared using this approach. The effect of *pH* change on the composite films tends to incline towards alkaline *pH* also indicated that these composite films will be most appropriate for suppurating wound dressing because the increase in *pH* by the wound dressing aids in neutralising the surrounding microenvironment and thus helps in healing^21^. For any wound healing applications, it is desirable that wound area is maintained moist to facilitate the healing process with minimal scarring by absorbing the wound fluid which can stimulate the keratinocyte proliferation and fibroblast growth^22^. The time required by the film to deform by absorbing the simulated wound fluid was calculated as the deformation time. The simulated wound fluid was prepared using gelatin and water and the deformation time (time required for swelling) was found to be approximately 5 min for the hydrophobically modified alginate-Psyllium husk composite films.

Adsorption of serum-derived proteins by the wound dressing causes delay in the wound healing process due to increase adherence of dressings leading to trauma and acute pain during dressing changes. Therefore, lower protein adsorption by wound care films is preferred for rapid healing process^23^. For this reason, the composite films were subjected to protein adsorption study using bovine serum albumin. A linearity plot of UV (Ultra Violet) absorption of albumin was constructed by plotting the absorbance against different concentrations of albumin (Supplementary Table S15 and Fig. S9). A composite film was immersed in a selected concentration of albumin solution to adsorb the protein. The difference in the absorbance reading before and after immersion of the film into the bovine serum albumin solution was calculated to find out the total protein adsorption (Table 2). The films did not show significant protein adsorption which suggests that the films when applied on wound surface will easily come off.

**Table 2:** Protein adsorption by the Modified alginate-psyllium husk gel composite film.

Conditions	Absorbance	Concentration of protein (mg/ml)	Total Protein adsorbed (mg/ml))
Before immersion	0.4436	77.69	77.69−71.51 = 6.18
After immersion	0.4195	71.51	

**Figure 5:**
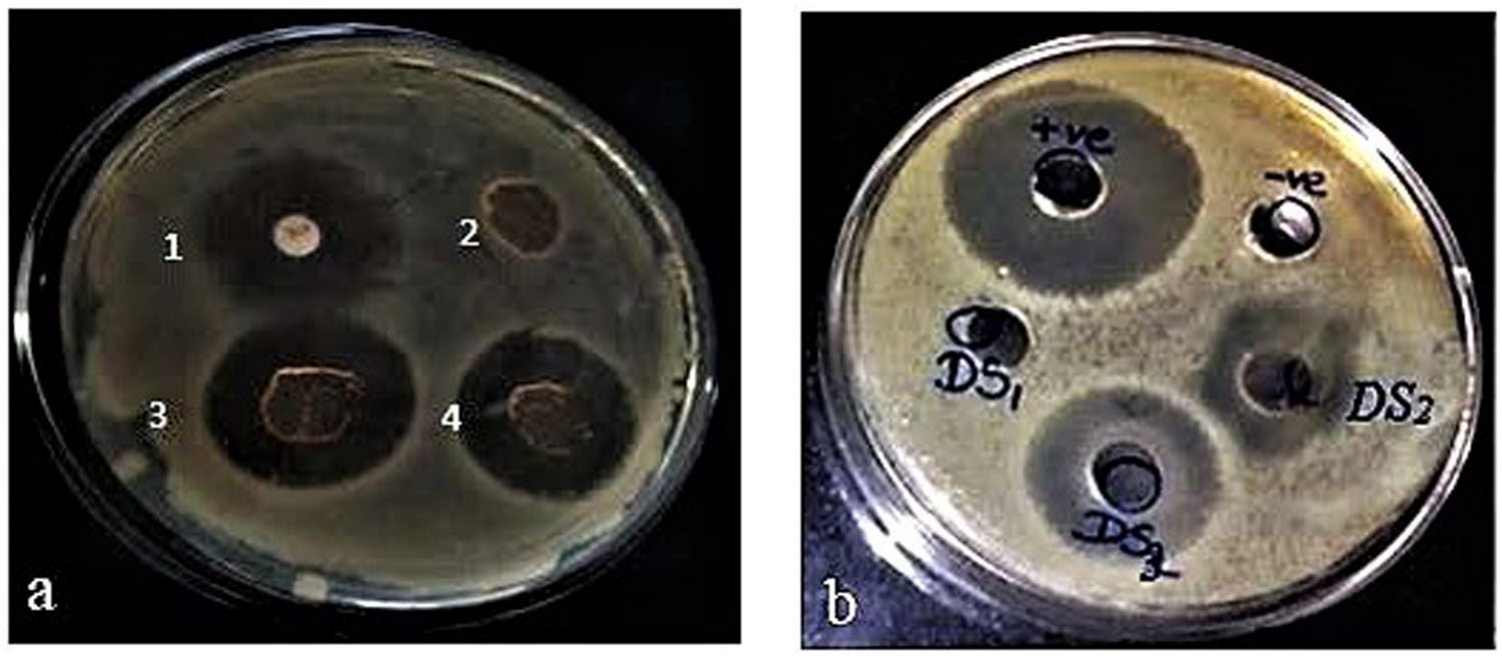
(**a**) Zone of growth inhibition of 1. antibiotic disc 2. psyllium husk gel-alginate composite film 3. & 4. lauroyl grafted alginate-psyllium husk gel composite film against *Escherichia coli* (**b**) Zone of growth inhibition of the lauroyl grafted alginate at various concentrations against *Escherichia coli*.

Finally, the synthesized glycolipid as well as glycolipid-*Psylluim* husk gel composite film was subjected to antimicrobial study against *Escherichia coli*, the common microbial contaminant of *Psyllium* husk, to verify the efficacy of the film against microbial contaminations^24^. The composite film containing the lauroyl grafted alginate displayed a strong zone of inhibition compared to the standard antibiotic streptomycin while films without glycolipid failed to show any antimicrobial property (Table 3 and Fig. 5a).

**Table 3:** Zone of growth inhibition of different films against *Escherichia coli*.

Sl No	Type of film	Zone of inhibition (in mm)
1	Streptomycin disc (10 µg/mL)	11.6
2	Psyllium husk gel-alginate composite film	0
3	Modified alginate-psyllium husk gel composite film (first batch)	11.3
4	Modified alginate-psyllium husk gel composite film (second batch)	11.5

However, the film discs were found to be swollen after 24 hrs of incubation period because of the inherent swelling properties of the composite film. This result prompted us to investigate the antimicrobial effect of the lauroyl grafted alginate alone against *Escherichia coli*. The glycolipid was found to have an improved antimicrobial effect against *Escherichia coli* (Supplementary Table S16, Fig. 5b) of its own.

This result showed that the synthetic modification of alginate to its lauoryl grafted hydrophobic glycolipid counterpart not only imparts enhanced physicochemical and mechanical properties to the composite film but also renders it to have antimicrobial properties and lower protein adsorption properties.

To conclude the present study, the glycolipid containing composite film yielded excellent physicochemical and mechanical properties. The physicochemical properties indicated that the film can be prepared with desired thickness without much weight variations. The film displayed enhanced results in the folding endurance, percentage elongation, swelling studies, *in vitro* deformation studies, moisture vapour transmission rate, loss on drying and effect of *pH* change studies indicating that film can be an excellent wound dressing especially for suppurating wounds. Additionally, the composite film was also found to have a lower protein adsorption property which is desirable in wound dressing as it would make the dressing to come off easily from the wound surface during the changes. The antimicrobial property rendered by the lauoryl grafted glycolipid derivative of alginate was found to be an added asset as it will not only wade off the microbial load of the film during storage but also will be helpful in the biomedical utility of the composite film including wound dressing.

## Experimental Methods

### Materials

*Psyllium* husk was purchased from local vendor (Laxmi brand, Unjha). Lauric acid and bovine serum albumin was purchased from SD Fine Chemical Ltd. (Mumbai, India). Sodium alginate (average molecular weight of ~60,000 g/mol), triethanolamine and thionyl chloride were obtained from Loba Chemie Pvt Ltd (Mumbai, India). All solvents were purchased from Research Lab (Mumbai, India) and were distilled, dried and purified according to the standard procedures prior to use. The reactions were monitored using precoated thin layer chromatography plates purchased from Merck India (Mumbai, India). UV readings were taken on a Perkin-Elmer LAMBDA-25 double beam spectrophotometer (Perkin-Elmer Inc., USA) supplied with matched square cuvettes of 1.0 cm light path length. Infrared spectra (wavenumbers in cm^−1^) were recorded on Perkin-Elmer RX1 FTIR spectrophotometer (Perkin-Elmer Inc., USA) model using potassium bromide pellets. Viscosity was measured on a Brookfield DV-III Ultra viscometer (Brookfield, USA) whereas DSC was recorded on a Mettler Toledo DSC 1 Star System (Mettler Toledo, Switzerland). Additionally, the procured sodium alginate, *Psyllium* husk and lauric acid were characterised for their authenticity of the label claim as per the monographs described in Indian Pharmacopoeia 2014 and USP 35 (United States Pharmacopoeia 35). The details are provided in the supplementary characterization data.

### Synthesis of lauric acid grafted alginate (glycolipid)

A mixture of lauric acid (0.8 g, 4 mmol) and thionyl chloride (10 mL) was stirred at room temperature for 3 h. The excess of thionyl chloride was vacuum evaporated to obtain lauroyl chloride. The chlorinated residue was added drop wise with stirring to a solution of sodium alginate (3 g, 1 mmol) in triethanolamine (10 mL) to obtain a jelly like substance. It was kept for overnight stirring at room temperature. The obtained solid was filtered, washed several times with distilled water to remove excess of unreacted lauroyl chloride and dried to yield the lauroyl esters of alginic acid (3.8 g) as a yellowish white powder. The esterification of hydroxyl groups of alginate was confirmed by FTIR spectra. Purity was checked by performing Lasaigne’s sodium fusion extract test to rule out presence of nitrogen and change in DSC thermogram to confirm absence of starting materials.

### Extraction of Psyllium husk mucilage

#### Hot extraction

Accurately weighed *Psyllium* husk (5 g) was grinded in a mortar, hot distilled water (100 mL) was added to it and stirred on a hotplate cum magnetic stirrer at 25 RPM and 90 ± 1 °C for about 40 min. The mixture was filtered through a clean muslin cloth to get the gel.

#### Cold extraction

Accurately weighed *Psyllium* husk (5 g) was grinded in a mortar, distilled water (100 mL) was added to it, soaked overnight and stirred on a magnetic stirrer at 25 RPM for about 40 min at room temperature. The mixture was filtered through a clean muslin cloth to get the gel.

#### Acid-base extraction

Accurately weighed *Psyllium* husk (5 g) was grinded in a mortar, dilute NaOH was added (100 mL, 1% v/v) to it. Solution was stirred for 5 hrs until a viscous solution was obtained. Dilute hydrochloric acid (10%) was added with vigorous stirring until it was neutral to pH paper. The mucilage was filtered through muslin cloth to obtain the hydrolysed gel.

### Determination of viscosity of gel obtained by hot extraction method

The gel obtained by hot extraction process from the *Psyllium* husk was subjected to rheometry studies before and after sterilization. The gel of different concentrations (1, 2, 3, 4, 5 & 6% w/v) was prepared and the viscosity was measured using Brookfield viscometer and spindle No. 18. The gel was sterilized using an autoclave at 15 lbs pressure, 121 °C for 15–20 mins.

### Qualitative tests to identify the nature of carbohydrates present in the gel

The gel obtained by hot extraction method was subjected to qualitative test to confirm the presence of carbohydrate. For this purpose the gel was subjected to Molisch’s test, Benedict’s test and Seliwanoff’s test as described in the literature and the detailed protocol is given in the supplementary data^25^.

### Preparation of the hydrophobically modified alginate-Psyllium husk composite films

The lauric acid grafted alginate-*Psyllium* composite film was prepared using solvent casting method. Sodium alginate (0.75 g) was dispersed in hot distilled water (15 mL, 80 °C) with constant stirring to get a uniform dispersion. This solution was mixed with *Psyllium husk* gel (10 mL) maintained at 80 °C. To this mixture, the synthesized glycolipid derivative of alginate (1.75 g) was added and the solution was stirred to get uniform mixture. Zinc chloride (0.25% w/v) was incorporated to this mixture as a cross-linker. The solution was poured onto petri-plates (diameter 79 mm) and kept for drying at 80 °C for 3 h. Dried films were peeled-off and evaluated for their mechanical and physicochemical properties. Similarly, films comprising only *Psyllium husk* gel and alginate-*Psyllium husk* gel using either the cross linker or without the cross linker were studied for comparative purposes taking them as control against the lauric acid grafted alginate-*Psyllium* composite film.

### Evaluation of the films for their physicochemical properties, mechanical and performance indicators

#### Thickness measurement

The films of varying concentrations (1% w/v to 10% w/v) were measured using a hand-held digital micrometer (Mitutoyo South Asia Pvt. Ltd.) at the nearest ±1 μm. Each film was cut into circular dimension of 2.4 cm diameter and three measurements were taken at different locations (top, centre, and bottom) of each film. The means thickness of the three measurements of each film was noted.

#### Weight variation

Films of area 4.5 cm^2^ were cut from three different regions (upper, middle and lower) and weighed using analytical balance and the weights were recorded for the weight variation.

#### Folding endurance

Films of size 2 × 2 cm were cut using sharp blade and the folding endurance was determined by repeatedly folding it at the same place till a crack appeared. The number of times, the film could be folded at the same place without breaking gave the value of folding endurance.

#### Percentage elongation

The films were cut into strips from three different regions (upper, middle and lower) in triplicates and were stretched till it breaks. The difference in initial and final length were recorded and the percentage elongation was calculated as follows

$${\rm{Percentage}}\,{\rm{elongation}}=\{({\rm{Final}}\,{\rm{length}}-{\rm{initial}}\,{\rm{Length}})/{\rm{Final}}\,{\rm{Length}}\}\times 100$$

#### Moisture vapour transmission rate (MVTR)

The films were evaluated for moisture vapour transmission rate according to the ASTM (American Society for Testing and Materials) method. Circular films of 4.5 cm^2^ were cut and attached to the surface of the vial using adhesive. The films were fixed over the brim of the vial containing 2 g of silica as desiccant. Vials were weighed and kept in desiccators at 37 °C and high humidity condition controlled with saturated solution of potassium chloride. After appropriate time intervals (0, 5, 10, 15, 30, 45, 60, 90,120, 240, 1080, 1440 and 2880 mins) the vials were removed and weighed. The differences in weight of the films were calculated at each time intervals and water gain/cm^2^ was calculated as the indicator for moisture vapour transmission rate using the formula as given below.

$${\rm{Water}}\,{\rm{gain}}/{{\rm{cm}}}^{2}=({\rm{Final}}\,{\rm{weight}}-{\rm{initial}}\,{\rm{weight}})/4.5\,{{\rm{cm}}}^{2}$$

#### Swelling studies

The films were placed in Petri plates containing 4 mL of phosphate saline buffer (pH 7.4). The films were removed and weighed after an interval of 5 mins for a period of 1 hr and the increase in weight was calculated.

#### In vitro deformation studies

A gelatin solution of 4% w/v was prepared by heating gelatin (4 g) in distilled water (100 mL) and 20 mL of the solution was poured into a petri plate and allowed to set at 4 °C using a refrigerator. Three films of 2 cm^2^ were placed in the centre of the petri plate on top of the gelatin surface. The time required for the films to deform and swell was recorded as *in vitro* deformation time. Since the films absorbed water from the medium, the increase in diameter (D_t_) as a function of time was recorded against the initial diameter (D_i_) of the film. The swelling index was calculated using the formula:

$${\rm{Swelling}}\,{\rm{index}}=[({{\rm{D}}}_{{\rm{t}}}-{{\rm{D}}}_{{\rm{i}}})\div{{\rm{D}}}_{{\rm{i}}}]\times 100$$

The diameter was measured four times at 45 minute intervals and the mean was calculated. The experiment was performed in triplicate with fresh gelatin media.

#### Effect of pH

Phosphate buffers of *pH* 4, phosphate saline buffer of pH 7.4 and phosphate buffer of *pH* 7.5 were prepared and 4 mL of the individual solutions were kept in different Petri plates. The films were placed and the changes in *pH* were recorded at appropriate time (0, 5, 10, 15, 20, 30, 40, 45, and 50 mins) intervals.

#### Protein adsorption studies

The standard curve of UV absorbance was plotted by taking various concentrations (20, 40, 60, 80, 100 mg/ml) of bovine serum albumin solutions in distilled water against their respective absorbances obtained at λ_max_ of 280 nm. A bovine serum albumin solution of 60 mg/ml concentrations was prepared and its absorption was determined. The film of 1 cm^2^ was cut and immersed in 10 ml phosphate saline buffer of pH 7.4 and were incubated at 37 °C for 1 hr until maximum swelling was obtained. This swelled piece of film was transferred to the buffer solution containing 60 mg/ml bovine serum albumin and shaken for 4 hr at 37 °C using a mechanical shaker to allow the protein adsorption by the film. The film was removed and protein adsorption was calculated by determining the difference in the absorbance reading before and after immersion in to the bovine serum albumin solution.

#### Anti-microbial evaluation of the films

The anti-microbial activity of the film was evaluated on *Escherichia coli* MTCC (Microbial Type Culture Collection and Gene Bank) no. 1687, ATCC (American Type Culture Collection) no. 25922. Petri-plates, nutrient agar media, soyabean casein digest broth and other required accessories were autoclaved at 121 °C at 15 psi for 15 mins. The test organism concentrations were set to 10^8^ CFU (Colony Forming Unit) using McFarland dilution method. For anti-microbial evaluation of films, disc diffusion method was used. The disc containing 10 µg/mL of streptomycin was used as the standard. The microbial culture was spread on to the solidified agar media containing soyabean casein digest broth using a glass spreader. Films were cut in circular disc of 8 mm diameter and were placed on to the agar media and were incubated for 24 hrs at 32.5 ± 2.5 °C. After 24 hr, the zone of inhibition was observed.

#### Statistical Analysis

The experiments were performed in triplicate, and data were expressed as the means ± standard deviations. The statistical analysis was done by using Graph Pad Prism statistical software package 5.

## Electronic supplementary material

Suplementary Data
