# Author Correction: Preparation of Lauroyl Grafted Alginate-Psyllium Husk Gel Composite Film with Enhanced Physicochemical, Mechanical and Antimicrobial Properties

**DOI:** 10.1038/s41598-019-55233-4

**Published:** 2019-12-04

**Authors:** Clara Fernandes, Pratap Chandra Acharya, Shikha Bhatt

**Affiliations:** 1 Shobhaben Pratapbhai Patel School of Pharmacy and Technology Management, SVKM’S NMIMS, Mumbai, 400056 India; 2 Department of Pharmacy, Tripura University (A Central University), Suryamaninagar, 799022 Tripura (W) India

Correction to: *Scientific Reports* 10.1038/s41598-018-35632-9, published online 21 November 2018

In the original version of this Article, Clara Fernandes was omitted as a corresponding author. Correspondence and request for materials should also be addressed to clara_fern@yahoo.co.in. This error has now been corrected in the HTML and PDF versions of the Article, and in the accompanying Supplementary Information file.

