# Supplementary material for: Preparation of Lauroyl Grafted Alginate-Psyllium Husk Gel Composite Film with Enhanced Physicochemical, Mechanical and Antimicrobial Properties
Source: Sci Rep. 2018 Nov 21;8:17213. doi: 10.1038/s41598-018-35632-9 (PMC6249254; doi:10.1038/s41598-018-35632-9)
Supplement: Supplementary file 1 — Suplementary Data [file 41598_2018_35632_MOESM1_ESM.pdf]

PREPARATION OF LAUROYL GRAFTED ALGINATE-PSYLLIUM HUSK GEL COMPOSITE FILM WITH ENHANCED PHYSICOCHEMICAL, MECHANICAL AND ANTIMICROBIAL PROPERTIES

Clara Fernandes,[#1] **Pratap Chandra Acharya,**[*1,2] **& Shikha Bhatt**[1]

[1]*Shobhaben Pratapbhai Patel School of Pharmacy and Technology Management, SVKM'S NMIMS, Mumbai-400056, India*

[2]*Department of Pharmacy, Tripura University (A Central University), Suryamaninagar-799022, Tripura (W), India*

[*]Corresponding author. Tel.: +91 381 237 9406; Fax: +91 381 237 4802.

E-mail: pratap.aacharya@gmail.com

[#] Corresponding author. Tel.: + 91-22-26670871; Fax: + 91-22-26670816.

E-mail: clara_fern@yahoo.co.in

SUPPLEMENTARY CHARECTERIZATION DATA

1. Characterization of *Psyllium* husk

1.1 Identification of Ispaghula husk and seed (IP 2014)

Ispaghula husk and seed were mounted in cresol and examined under microscope. The seed and husk were mounted in ethanol and irrigated with water and were observed in microscope. Seed and husk were also mounted in 0.005 M iodine and were examined under motic microscope.

1.2 Swelling power of Ispaghula husk and seed (IP 2014)

Accurately 1 g of Ispaghula husk/seed was weighed and was transferred to 100 ml stoppered cylinder containing 90 ml of water, which was shaken well for 30 seconds and allowed to stand for 24 hour, shaking gently on 3 occasions during this period. Then sufficient amount of water was added to produce 100 ml, mixed gently for 30 seconds, avoiding air entrapment, allowed to stand for 5 hour and volume of mucilage produced was measured. The same procedure was repeated thrice.

1.3 Total ash of Ispaghula husk and seed (IP 2014)

Accurately 2 to 3 g of husk/seed was weighed in a tarred silica crucible and was incinerated, gently at first and then temperature was increased to 675±25 °C , until it got free from carbon, then cooled and weighed. If carbon free ash not obtained then charred mass was filtered with hot water and insoluble residue was incinerated at 675±25° C. After which it was cooled in a desiccator and ash was weighed and % total ash was calculated.

1.4 Acid insoluble ash of Ispaghula husk and seed (IP 2014)

Ash was boiled and with 25 ml of 2 M Hydrochloric acid for 5 minutes, the insoluble matter was collected and washed with hot water. Then cooled in a dessicator and weighed, % acid-insoluble ash was calculated.

1.5 Loss on drying of Ispaghula husk and seed (IP 2014)

Accurately 0.5 g of Ispaghula husk/seed was weighed and was transferred to a previously weighed porcelain dish which was weighed and dried in an oven at 105° C for 5 hour. After which it was weighed and loss on drying was calculated.

1.6 Heavy metals test of Ispaghula husk and seed (USP 35)

Method II

Preparation of acetate buffer

Accurately 25 g of ammonium acetate was weighed and was dissolved in 25 ml of water. 38 ml of 6 N Hydrochloric acid was added to adjust pH to 3.5 and was diluted with water to make 100 ml.

Procedure

Accurately 2 g of Ispaghula husk/seed was weighed and was transferred to a crucible and sufficient concentrated sulphuric acid was added to wet the substance. Ignited at low

temperature till it got completely charred after which 2 ml of nitric acid and 5 drops of concentrated sulphuric acid was added. It was heated until no fumes evolved and ignited in muffle furnace at 525° C until carbon completely burned off. Cooled, 4 ml of 6 N Hydrochloric acid was added and kept on a steam bath for 15 minutes. The residue was moistened with 1 drop of hydrochloric acid and filtered with 10 ml hot water and pH was adjusted between 3 and 4. Filtered if necessary and the filtrate were combined. 2 ml of pH 3.5 acetate buffer was added to each test tube containing standard and test preparation and 1.2 ml of Thioacetamide glycerine base TS was added. After which it was diluted with 50 ml of water and was allowed to stand for 2 minutes.

1.7 Microbial Enumeration Test

The test described hereafter will allow quantitative enumeration of bacteria and fungi that may grow under aerobic conditions. The tests are designed primarily to determine whether a substance or preparation complies with an established specification for microbiological quality.

Carry out determinations under conditions designed to avoid extrinsic microbial contamination of the product to be examined. The precautions taken to avoid contamination must be such that they do not affect any microorganisms that are to be revealed in the test.

Each of the bacterial species were grown separately in Casein Soyabean digest broth (Medium1) and incubated at 30°-35° for approximately for 18 to 24 hours. A*spergillus niger* was grown on Sabouraud dextrose agar at 20-25° C for 5-7 days.

Table S1: List of microorganisms and suitable growth medium

Microorganisms	ATCC No.	MTCC No.	Growth Medium	Incubation temperature (◦C)
Escherichia coli	25922	1687	Soyabean casein digest broth	32.5±2.5
Salmonella typhi	19430	98	Soyabean casein digest broth	32.5±2.5
Aspergillus niger	16888	144	Sabouraud dextrose broth	22.5±2.5

Preparation of Sample

Procedure for Non-fatty products insoluble in water : Psyllium husk was sieved through mesh No.60 and accurately 1 g Psyllium husk powder was weighed and dissolved in phosphate buffer solution pH 7.2 (i.e. 1 in 10 dilution was prepared). From the stock solution 1:100 and 1:1000 dilutions were prepared.

Preparation of Inoculum

For preparation of inoculums McFarland 0.5 standard is used as turbidity standard. The McFarland standard has particular application in preparation of bacterial inocula for performing the microbioal enumeration test. A sufficient amount of sterile saline TS was added to bacteria and fungi cultures and was diluted with sterile saline TS until its turbidity matched with that of McFarland 0.5 standard.

McFarland 0.5 standard

McFarland 0.5 standard was prepared by adding sulphuric acid (0.18 M) to an aqueous solution of barium chloride (0.048 M), which results in formation of a suspended barium sulphate precipitate.

Growth promotion by media

The already approved medium prepared from dehydrated medium or from the ingredients as a positive control.

Negative control To verify the test conditions, sterile buffered sodium chloride-peptone solution pH 7.0 was used as a negative control in place of test organism (inoculum).

Note- Negative control should not show any growth of microorganisms. If any growth is seen and negative control fails, its cause should be investigated.

Procedure:

Petri-plates, nutrient agar media and other required accessories were autoclaved at 121° C at 15 psi for 15 minutes. The test organism concentrations were set to 10^8 CFU using Mc Farland dilution method. In each petri plate 0.1 ml of 10^8 CFU mg/ml of microbial inocula and 1 ml of prepared sample was added. Afterwhich soyabean casein agar and sabaroud dextrose agar were added for bacterial and fungal species respectively.Preparation of test and control was done as follows:

Plate count method:

Surface-spread method

Petri dishes of 4” diameter were used, 15 ml of Casein Soyabean Digest agar was added, for cultivation of aerobic microorganisms or Sabouraud dextrose agar with antibiotic and for cultivation of fungi, at about 45°C to each Petri dish and allow to solidify. Plates were dried, in an incubator. Measured volume was spread, not less than 0.1 ml of the sample prepared as described earlier, over the surface of the medium. This method was used as it was easier to count the microbial colonies.

Interpretation of the results

The total aerobic viable count (TAC) is considered to be equal to the number of cfu found on Casein soyabean digest agar. If colonies of fungi are detected on this medium, they are counted as part of TAC. The total fungal count (TFC) is considered to be equal to the number of cfu found using Sabouraud dextrose agar with antibiotic.

Acceptance criteria for microbiological quality should be interpreted as follows –
10^1cfu : maximum acceptable count = 20
10^2cfu : maximum acceptable count = 200
10^3 cfu : maximum acceptable count = 2000 and so forth.

Table S2: Characterization of Ispaghula husk and seed

Sr. No.	Tests performed	Specification IP 2014	In house result		Inference
1	Identification test Cresol	When mounted in: Cresol-Transparent and angular particles with straight or curved edges	Cresol-Transparent and angular particles with straight or curved edges		It complies with the standard
	Ethanol	Ethanol- Mucilage in the outer part of epidermal cells swells rapidly and goes into solution	Ethanol- Mucilage in the outer part of epidermal cells swells rapidly and goes into solution		It complies with the standard
	0.005 M Iodine	0.005 M Iodine- Starch granules can be seen in some of the cells with thick walled, reddish brown endosperms	0.005 M Iodine- Starch granules can be seen in some of the cells with thick walled, reddish brown endosperms		It complies with the standard
2	Swelling power	The average of 4 determinations should not be less than 40 ml	For seeds Average volume = 43.75± 1 ml	For husk Average volume = 45.75± 1 ml	It complies with the standard
3	Total Ash	Ispaghula seeds and husk must contain not more than 4.5% for 1 g	%Total ash=4.1±1 %	%Total ash=4.3± 1%	It complies with the standard
4	Acid insoluble ash	Ispaghula seeds and husk must contain not more than 0.45% for 1g	%Acid-insoluble ash 0.31 ± 1%	%Acid-insoluble ash 0.37± 1%	It complies with the standard
5	Loss on drying	Must be not more than 12%,determined on 0.5g	6.85± 1% determined on 0.5 g	9.06± 1 % , determined on 0.5 g	It complies with the standard

6	Heavy metals test	Colour of test preparation should not be darker than standard preparation	Colour of Test preparation is not darker than Std prep	Colour of Test preparation is not darker than Std prep	It complies with the standard
7	Microbial enumeration test (According to USP)	Total combined molds and yeasts count doesn't exceed 1000 cfu/g	247 cfu/g	251 cfu/g	It complies with the standard

Inference: The procured psyllium husk was characterized as per IP 2014 and USP 35 and it complied with the pharmacopoeial limits.

2. Characterization of Sodium alginate (IP 2014)

2.1 Identification of Sodium alginate

Accurately 0.2 g of Sodium alginate was weighed and was dissolved in 20 ml of water with shaking and to 5 ml of resulting solution 1 ml of calcium chloride solution was added; to obtain a voluminous gelatinous precipitate.

2.2 Melting point

Melting point was determined using capillary method. Sodium alginate was filled in the capillary and one side was sealed and attached to thermometer. Melting point was observed to be greater than 300 ^{o}C.

2.3 Loss on drying

Accurately 0.2 g of Sodium alginate was weighed and was transferred to a previously weighed porcelain dish which was weighed and dried in an oven at 105° C for 4 hours. After which it was weighed and loss on drying was calculated.

2.4 Infrared Spectroscopy

Accurately 5 mg of Sodium alginate was weighed and 200 mg of KBr was added into mortar. The KBr pellet was previously dried in oven at 115° C for 1 hour and was triturated to form fine powder the disc was made with IR disc compressor and the disc was collected using Perkin Elmer FTIR in the range of wavelength from 400-4000 cm^{-1} and the background spectrum was collected using identical conditions.

Table S3: Characterization of Sodium alginate (IP 2014)

Sr. No.	Tests performed	Specification IP 2014	In house result
1	Identification test	Voluminous gelatinous precipitates should be produced	Voluminous gelatinous precipitates produced
2	Loss on drying	Not more than 15.0 %	LOD=11.5%

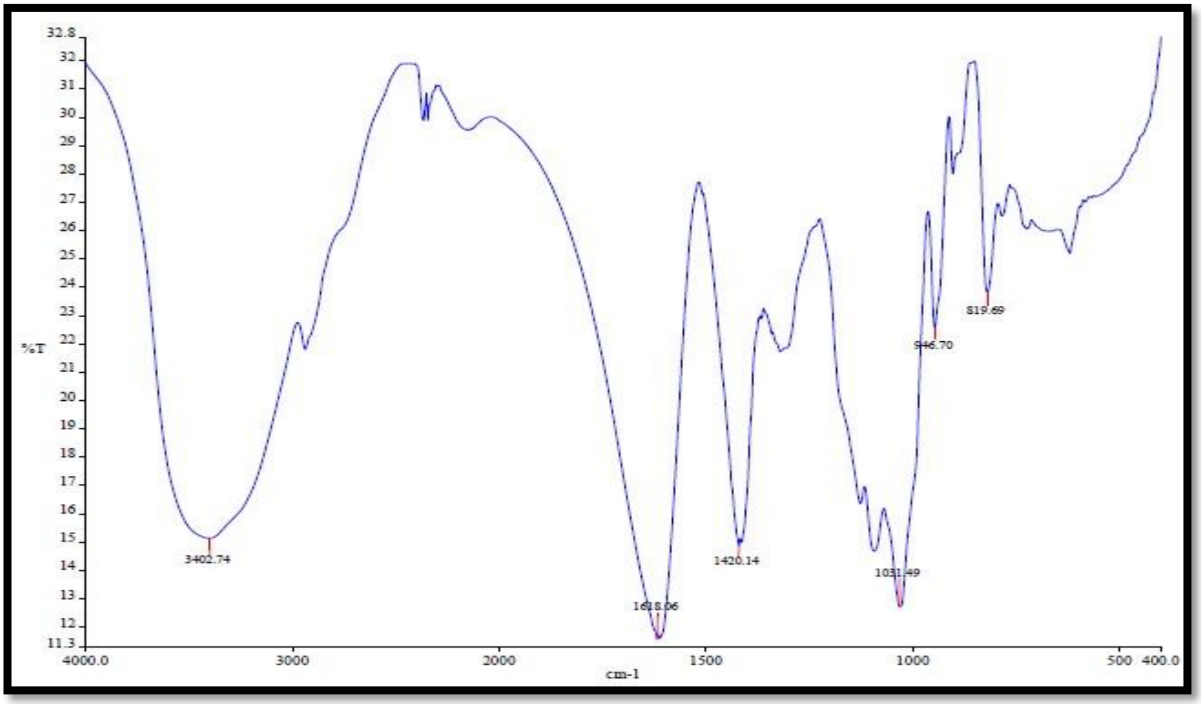

Fig S1: IR Spectra of Sodium alginate

Sodium alginate was characterized as per IP 2014 and compared with the pharmacopoeial monograph.

3. Characterization of lauric acid/Dodecanoic acid

3.1 Acid value

Accurately 0.5 g of Dodecanoic acid was weighed and was added in 25 ml of ethanol and 25 ml of ether. It was heated to dissolve and titrated with 0.1 N Potassium hydroxide using phenolphthalein as an indicator.

3.2 Saponification value

Accurately 3 g of Dodecanoic acid was weighed and was added in 50 ml of 0.5 N alcoholic solution of Potassium hydroxide in 250 ml conical flask. It was refluxed with shaking for 1 hour. Cooled and 1 ml of phenolphthalein was added as an indicator. Titrated with 0.5 N hydrochloric acid and blank reading was taken.

Inference

- Appearance: A white to pale yellow crystalline lump or powder
- Molecular weight: 200.32 g/mol
- Melting point: 45±1° C

Table S4: Characterization of Dodecanoic acid

Sr. No.	Tests performed	Specifications	In house results
1	Acid value	Acid value should be 252~287	264.9
2	Saponification value	Saponification value should be 253 -287	265.1

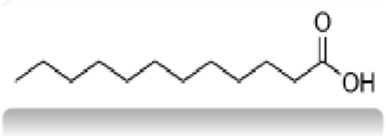

Fig S2: Chemical structure of Dodecanoic acid

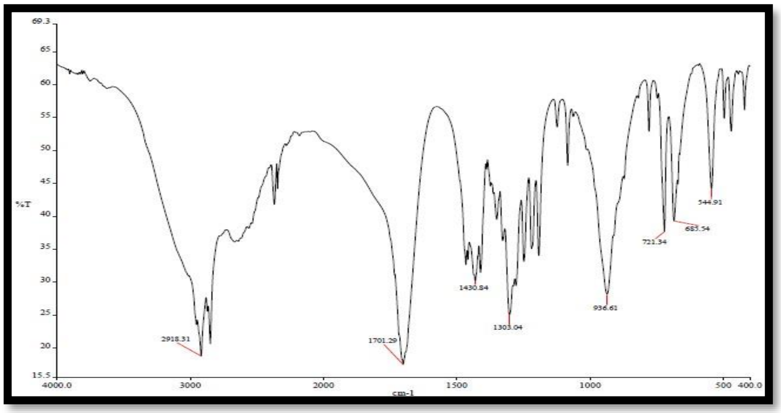

Fig S2: FTIR Spectra of lauric acid

4. Protocol for qualitative tests to identify nature of carbohydrates present in the gel

Molisch's Test

Accurately 1 ml of Ispaghula husk gel (any concentration of gel can be chosen) was weighed and was immersed in 10 ml of distilled water, this solution was boiled and to 5 ml of this solution, 2 drops of Molisch reagent and 3 ml of concentrated sulphuric acid were added and was observed for change in colour.

Benedict's Test

In 10 ml of distilled water accurately 1 ml of Ispaghula husk gel (any concentration of gel can be chosen) was immersed, this solution was boiled and to 1 ml of this solution, 5 ml of

Benedict’s reagent was added and was placed in water bath. Any colour change was observed.

Seliwanoff’s Test

Accurately 1 ml of Ispaghula husk gel (any concentration of gel can be chosen) was weighed and was immersed in 10 ml of distilled water, this solution was boiled and to 1 ml of this solution, 5 ml of Seliwanoff’s reagent was added. It was placed in water bath for 15 minutes. Any colour change was observed.

Table S5: Qualitative tests of carbohydrates in the psyllium husk gel

**Carbohydrate**	**Molisch’s test**	**Bendict’s test**	**Seliwanoff's Test**
Glucose	Brown colour	Red precipitate	**Gold colour**
Fructose	Dark brown colour	-	Blood red colour
Galactose	Red colour		Light yellow colour
Arabinose	**Dark red colour**	Amber colour	-
Ribose	Light brown colour	-	Dark yellow colour
Xylose	Brown colour	-	-
Sucrose	-	**No colour change**	Blood red colour
	**Arabinose present**	**Sucrose present**	**Glucose be present**

SUPPLEMENTARY TABLES

Table S6. Determination of viscosity of the psyllium gel by hot extraction method

Conc.	20 rpm		30 rpm		40 rpm		50 rpm		60 rpm		80 rpm		100 rpm	
(%w/v)	B	A	B	A	B	A	B	A	B	A	B	A	B	A
1	61.9	61.6	49.8	48.5	42.6	42.3	33.6	33.2	26.5	26.1	24.1	24.4	20.7	20.4
2	62.5	61.3	50.5	48.1	43.5	40.2	34.9	33.5	28.3	26.7	24.6	23.1	22.3	20.1
3	64.5	63.9	51.6	51.3	44.9	44.5	36.4	36.2	29.4	28.1	26.8	26.4	23.9	23.6
4	66.2	66.0	54.1	53.6	46.7	46.2	37.1	36.7	30.3	30.1	27.9	27.5	25.1	24.4
5	66.7	66.5	54.7	54.2	47.8	47.1	38.4	38.2	31.9	31.7	28.1	27.9	25.8	25.6
6	67.3	66.8	55.2	54.8	48.4	48.2	39.2	38.5	32.5	31.8	28.4	27.6	26.2	25.8

B= Before sterilization A= After sterilization

Table S7. Optimization trials to determine the effect of Ispaghula husk concentration and effect of loss on drying on the film formation

Concentration	Observation	% moisture content	Inference
1% w/v	No film formation	5.63	Concentration too low for film formation
2% w/v	Brittle film	7.89	
3% w/v	Brittle film	10.11	
4% w/v	Peelable film	13.56	Films with desired film forming properties were obtained
5% w/v	Peelable film	14.68	
6% w/v	Sticky film	25.91	No film formation
7% w/v	Uneven, viscous, thick	38.17	
8% w/v	Uneven, viscous, thick	46.91	
9% w/v	Uneven, viscous, thick	65.71	
10 % w/v	Uneven, viscous, thick	74.39	

*Drying time: 3 hrs, Temperature= 80 °C

Table S8. Physicochemical and mechanical parameters of Psyllium husk gel film

Concentration (in % w/v)	Thickness (in mm) study in triplicate	Weight variation (in g) study in triplicate	Folding endurance (in no. of times)	% Elongation study in triplicate
1	0.11 ±0.1	0.019±0.006	7±0.05	0.66±0.23
2	0.14±0.1	0.025±0.004	15±0.03	1.29±0.11
3	0.93±0.1	0.028±0.003	45±0.02	4.72±0.01
4	1.40±0.1	0.035±0.002	128±0.01	6.32±0.03
5	1.52±0.1	0.046±0.001	133±0.02	5.25±0.01
6	1.55±0.1	0.053±0.005	176±0.06	6.93±0.01
7	2.65±0.1	0.072±0.007	247±0.04	4.61±0.26
8	2.76±0.1	0.076±0.02	255±0.05	1.43±0.14
9	3.11±0.1	0.081±0.004	310±0.08	0.66±0.23

Table S9. Physicochemical and mechanical parameters of Psyllium husk gel-alginate composite film

Concentration of *Psyllium* husk gel and alginate (in % w/v)	Thickness (in mm) study in triplicate	Weight variation (in g) study in triplicate	Folding endurance (no. of times)	% Elongation study in triplicate
4.25	0.33±0.08	0.447±0.05	131.33±2.51	22.38±0.03
4.50	0.26±0.01	0.028 ±0.17	131.33±2.08	20.86±0.02
4.75	0.35±0.02	0.472 ±0.01	135.33±0.57	24.28±0.02
5.00	0.47±0.02	0.245 ±0.09	138.0±3.0	19.92±0.70
5.25	0.36±0.15	0.509 ±0.019	140.33±1.15	19.69±0.42
5.50	0.45±0.08	0.525 ±0.008	143.33±1.52	22.76±0.15

Table S10. Swelling studies and in vitro deformation of Psyllium husk gel-alginate composite film

Concentration of *Psyllium* husk gel and alginate (in % w/v)	% Swelling study in triplicate	*In vitro* deformation (in min) study in triplicate
4.25	9.43±0.12	5.1±0.23
4.50	16.63±0.21	6.3±0.15
4.75	14.30±0.02	5.5±0.01
5.00	25.71±0.11	4.7±0.16
5.25	27.67±0.09	5.4±0.24
5.50	29.67±0.15	5.2±0.30

Table S11. Result of MVTR of different optimised films

Time (in min)	MVTR of films made with		
	Psyllium husk gel (4 % w/v)	Psyllium husk gel-alginate (4.75 % w/v)	Modified alginate-psyllium husk gel (5 % w/v)
0	0	0	0
5	0.012	0.027	0.031
10	0.024	0.041	0.047
15	0.045	0.064	0.072
20	0.066	0.073	0.078
30	0.072	0.094	0.096
45	0.093	0.103	0.108
60	0.102	0.118	0.139
90	0.117	0.127	0.148
120	0.125	0.162	0.162
240	0.159	0.42	0.426
1080	0.417	0.562	0.569
1440	0.552	0.853	0.698
2880	0.697	0.717	0.72

Table S12. Results of loss on drying of different optimized films

Time (in min)	Loss on drying (drying rate in g/min) of films made with		
	**Psyllium husk gel (4 % w/v)**	**Psyllium husk gel-alginate (4.75 % w/v)**	**Modified alginate-psyllium husk gel (5 % w/v)**
0	0	0	0
5	0.102	0.126	0.050
10	0.115	0.089	0.063
20	0.157	0.1295	0.050
30	0.154	0.113	0.068
40	0.130	0.129	0.071
50	0.115	0.120	0.065
60	0.127	0.112	0.068
120	0.073	0.059	0.038
240	0.045	0.036	0.023
1080	0.01	0.0089	0.0056
1440	0.008	0.0069	0.0047

Table S13. Effect of change in pH of the optimized films in phosphate buffer (pH 7.4)

Time (min)	Psyllium husk gel (4 % w/v)	Psyllium husk gel-alginate (4.75 % w/v)	Modified alginate-psyllium husk gel (5 % w/v)
0	7.4	7.4	7.4
5	7.41	7.4	7.41
10	7.39	7.39	7.39
15	7.39	7.38	7.38
20	7.37	7.35	7.41
30	7.36	6.91	7.43
40	7.36	6.89	7.42
45	7.36	6.89	7.42
50	7.36	6.89	7.41

Table S14. Effect of change in pH of the optimized films in phosphate buffer (pH 4)

Time (min)	Psyllium husk gel (4 % w/v)	Psyllium husk gel-alginate (4.75 % w/v)	Modified alginate-psyllium husk gel (5 % w/v)
0	4	4	4
5	4	4	4.11
10	4.11	4.1	4.16
15	4.13	4.18	4.29
20	4.15	4.21	4.31
30	4.17	4.21	4.33
40	4.16	4.21	4.32
45	4.19	4.21	4.31
50	4.19	4.21	4.31

Table S15. UV absorbance of bovine serum albumin at different concentrations

Concentration (mg/ml)	Absorbance (λ_{max} 280 nm)
20	0.2280
40	0.2893
60	0.3585
80	0.4638
100	0.5295

Table S16. Zone of growth inhibition of the lauroyl grafted alginate against Escherichia coli

Sample code	Sample name	Concentration (in µg/mL)	Zone of inhibition (in mm)
-ve	Negative control	0	0
+ve	Antibiotic	100	10.3
DS_1	Lauroyl grafted alginate	100	0
DS_2	Lauroyl grafted alginate	300	4.5
DS_3	Lauroyl grafted alginate	500	9.7

SUPPLEMENTARY FIGURES

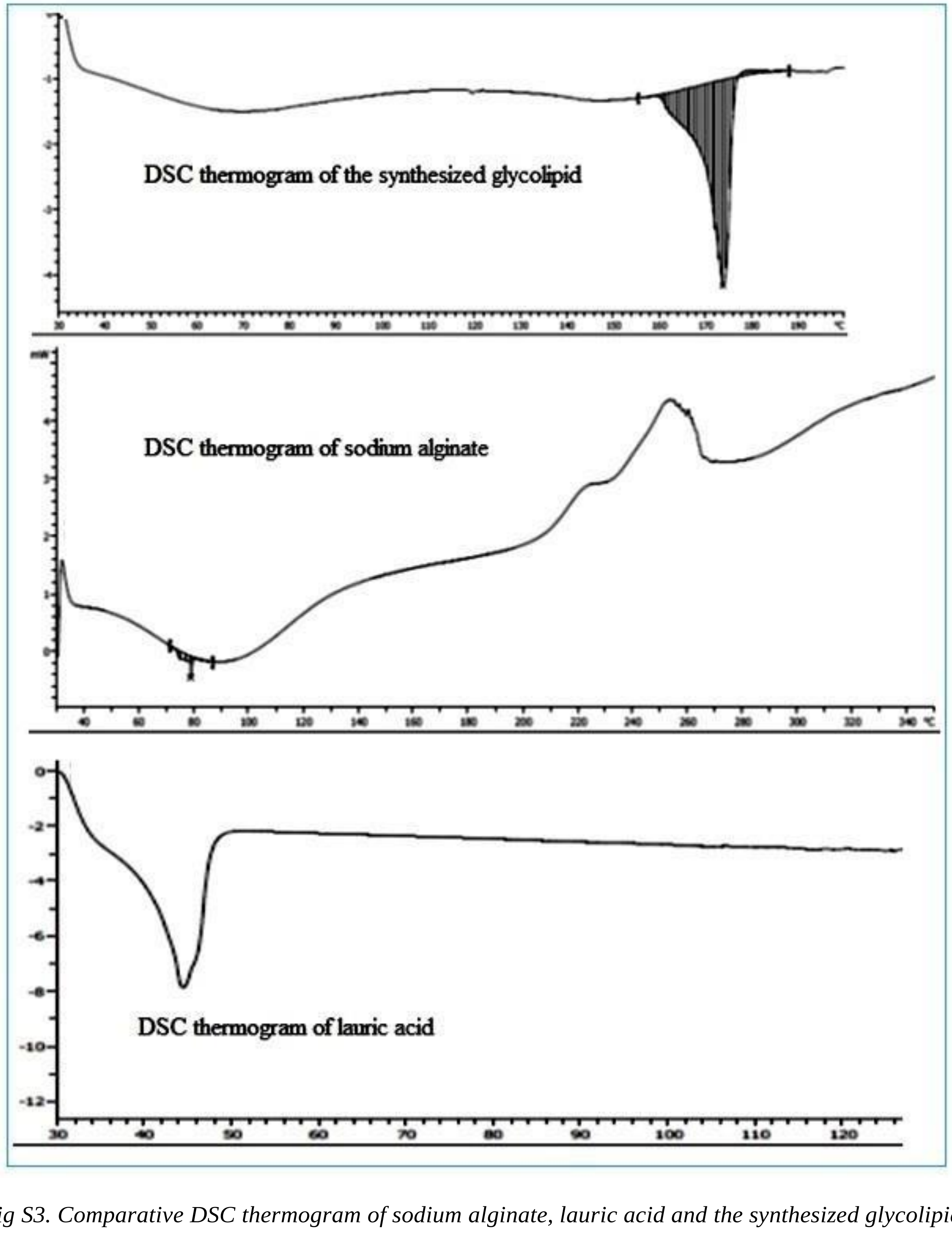

Fig S3. Comparative DSC thermogram of sodium alginate, lauric acid and the synthesized glycolipid

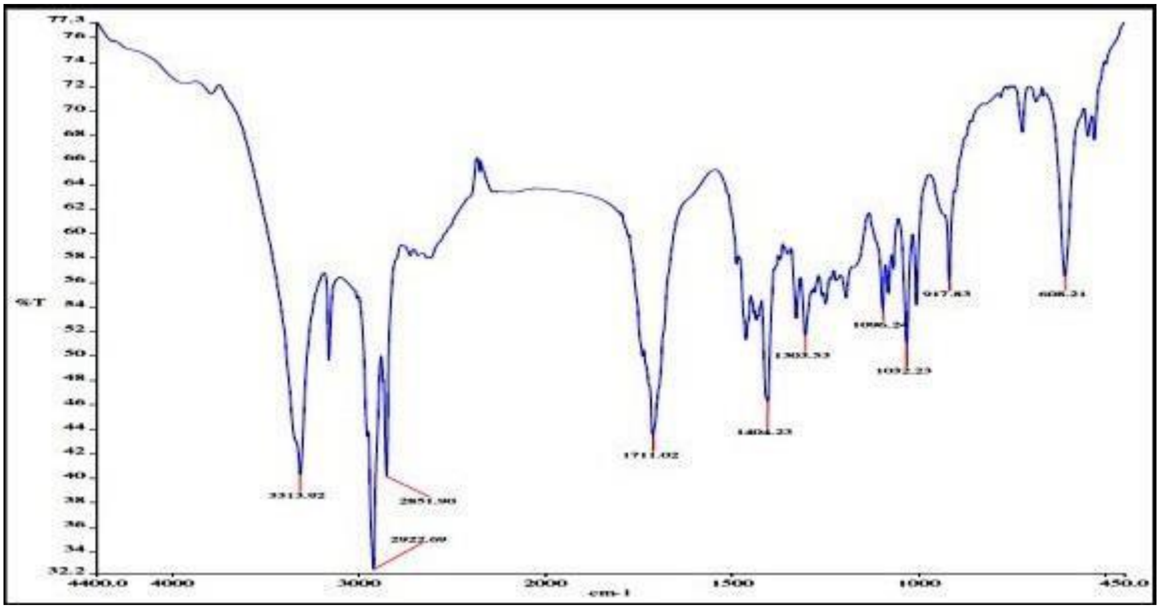

Fig S4. FTIR spectrum of the synthesized glycolipid ***(3)***

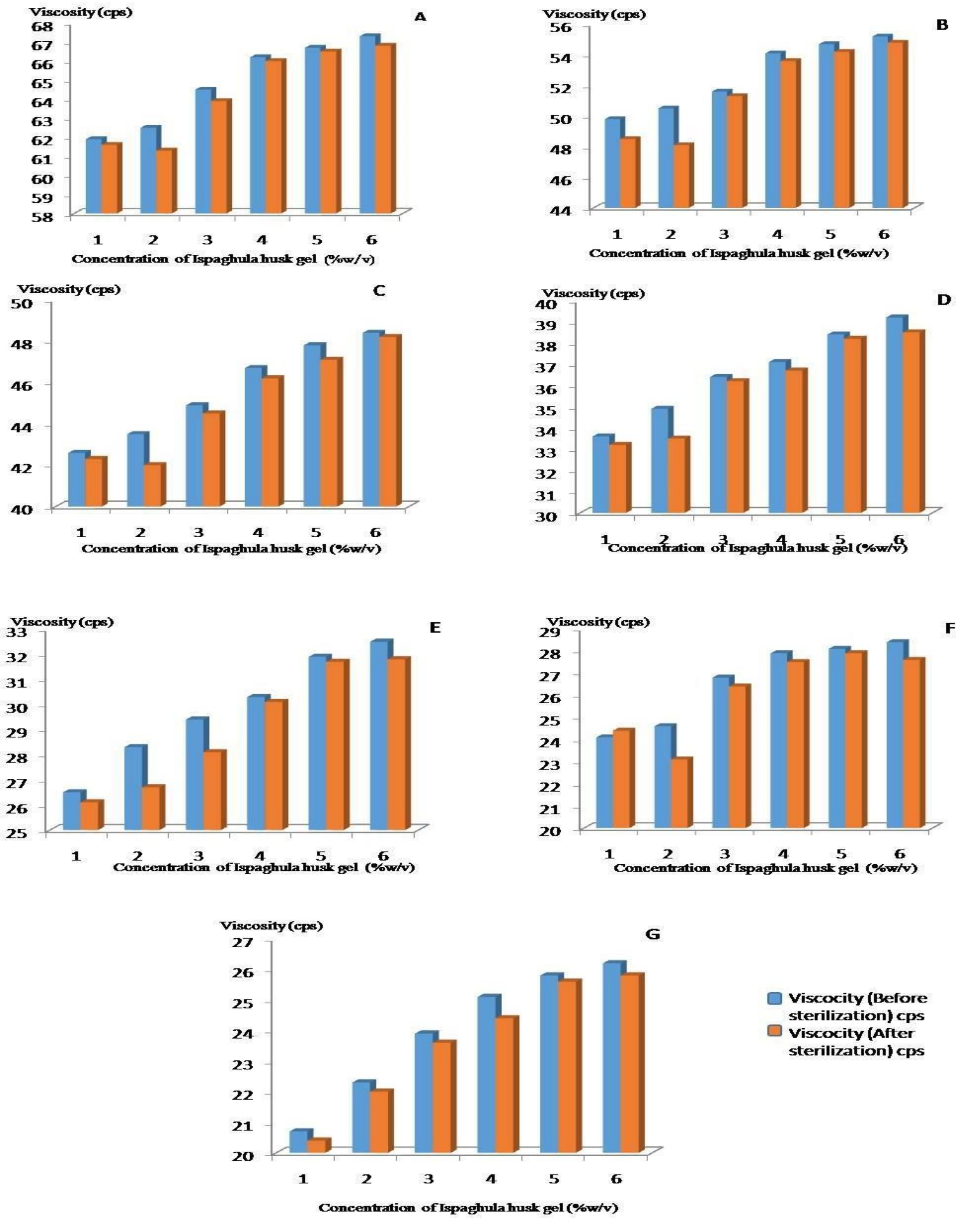

Fig S5. Viscosity of psyllium gel at (A) 20 rpm (B) 30 rpm (C) 40 rpm (D) 50 rpm (E) 60 rpm (F) 80 rpm and (G) 100 rpm before and after sterilization

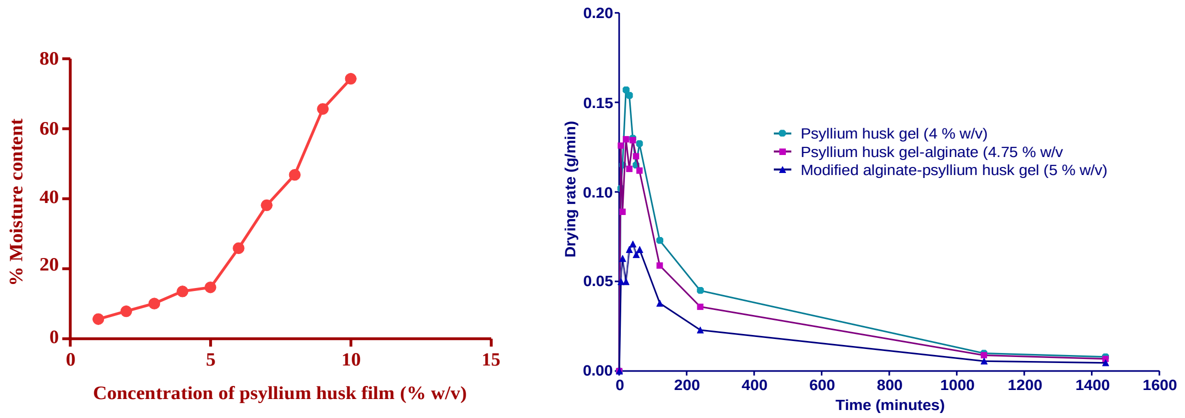

Fig S6. (a) Loss on drying of Psyllium husk film at 80 ^{0}C (b) Comparison of loss on drying of the optimized composite films

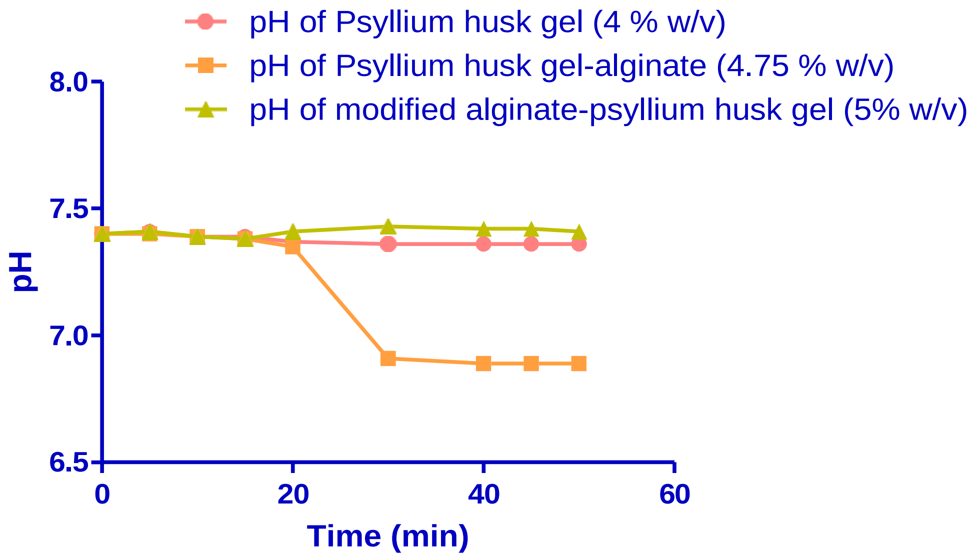

Figure S7. Comparison of change in pH of the composite films in phosphate buffer pH 7.4

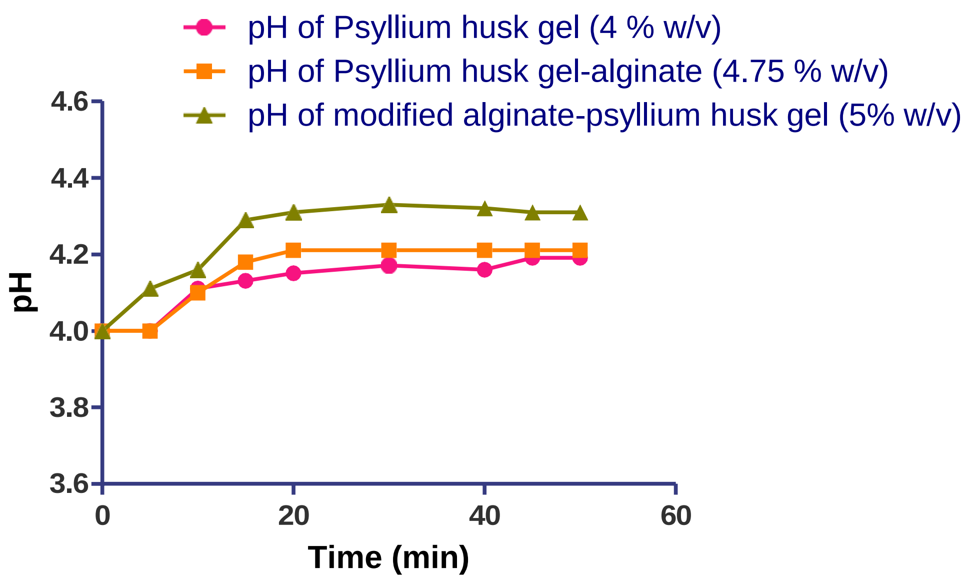

Figure S8. Comparison of change in pH of the composite films in phosphate buffer pH 4

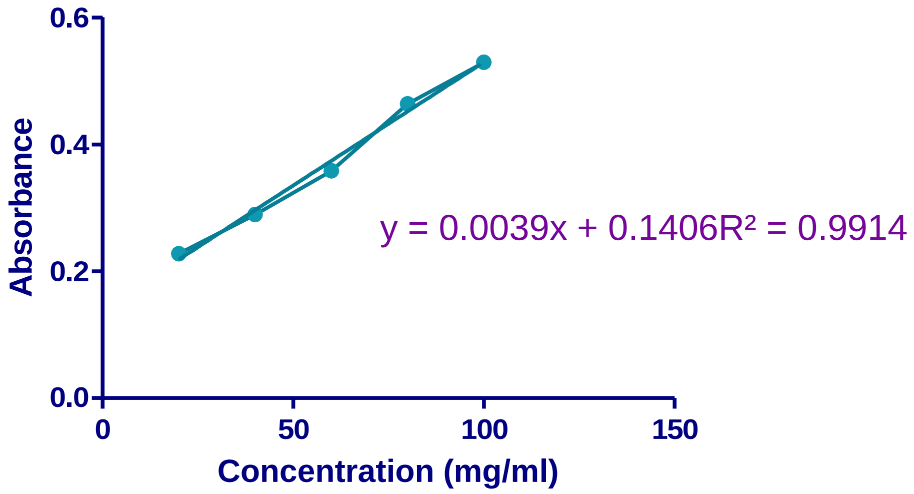

Figure S9. Linearity plot of absorbance of bovine serum albumin
